# Implementation of My Hearing PREM Into Three UK Audiology Services: A Pluralist Approach to Planning, Design and Evaluation

**DOI:** 10.1111/hex.70659

**Published:** 2026-04-11

**Authors:** Amanda Hall, Helen Pryce, Georgina Burns‐O'Connell, Sian Smith, Sian Noble, Jon Banks

**Affiliations:** ^1^ School of Optometry, Department of Audiology, College of Health and Life Sciences Aston University Birmingham West Midlands UK; ^2^ Children's Hearing Centre, St Michael's Hospital University Hospitals Bristol and Weston NHS Foundation Trust Bristol Avon UK; ^3^ Population Health Sciences, Bristol Medical School University of Bristol Bristol Avon UK; ^4^ National Institute for Health Research, Applied Research Collaboration West (NIHR ARC West) University Hospitals Bristol and Weston NHS Foundation Trust Bristol Avon UK

**Keywords:** audiology, hearing loss, hearing therapy, implementation, patient experience, patient reported experience measure, person based approach, PREM

## Abstract

**Introduction:**

My Hearing PREM (patient reported experience measure) is a validated tool for use in audiology services, to measure patients' experiences of living with hearing loss, and support from family and services. This study planned and evaluated the process of implementing My Hearing PREM into audiology services.

**Methods:**

Implementation planning and design was informed by the person‐based approach and involved patients, public and audiology staff. My Hearing PREM was implemented into three NHS audiology services for 3 months. Evaluation of implementation used quantitative methods to determine PREM use and cost, and qualitative interviews with staff and patients to assess the implementation process.

**Results:**

A total of 95 participants contributed data across planning to evaluation. Clinicians were generally willing to use the PREM and it was seen as a tool to access the lived experience of patients. Patients felt the PREM enabled them to reflect on their situation and experiences. The per patient cost of PREM implementation was between £1.57 and £3.18. PREM use varied across sites and integration into routine practice was inconsistent. Barriers to use within services included time constraints, difficulties in storing responses on electronic systems, discomfort from staff asking patients about their perceptions of clinical care, and views that it duplicated work.

**Conclusions:**

My Hearing PREM can support, at low cost, more personalized, reflective care in audiology by bringing patient experience to the fore. Long‐term integration in clinical services will depend on tackling practical barriers as well as addressing clinician concerns about the purpose of PREMs and aligning with services' ways of working.

**Patient or Public Contribution:**

A PPIE representative with lived experience of hearing loss contributed throughout. They contributed to the development of interview schedules, reviewed data analysis interpretations to ensure findings reflected participants experiences, and provided guidance on the development of implementation resources. This collaborative approach ensured that the implementation planning remained grounded in the real‐world experiences and need of people with hearing loss.

## 1 Introduction

Patient reported measures are routinely used in healthcare and can be seen as part of an increasing move to recognize individual patient reporting of their health as a legitimate source of information [1, 2]. Understanding patient experience is required to facilitate care in line with patient values and preferences [3], with positive experiences of healthcare linked to improved patient safety and clinical effectiveness across a range of conditions [4]. While patient reported outcome measures (PROMs) are tools to measure health status in a standardized format [5], patient reported experience measures (PREMs) measure the experience of care and/or the experience of living with a health condition [6]. They can be used to inform individual clinical care as well as provide markers of quality at the organizational and structural level of healthcare [6]. Their use can drive quality improvement (QI) activities within services [6, 7, 8].

Within audiology services, capturing patient experience is essential to understand how hearing loss and interventions impact an individual's daily life beyond clinical measures, that is, a move from a medical to a lifeworld model of care where the patient is considered holistically [9]. The current paper forms part of a larger research program focused on patient experience of hearing loss—the Hearing Loss and Patient Reported Experience (HeLP) research study (award ID: NIHR131597). The protocol for the HeLP program of work was published in 2023 [10], with involvement and engagement of patients, their caregivers, public, audiology clinicians and key stakeholders planned and included throughout the whole program of work [11]. The first phase of the program involved developing a conceptual model of ‘the lived experience of hearing loss’ through theoretical modelling and from inductive data [12, 13]. This model formed the basis for development of a prototype PREM which was refined using factor analysis and RASCH analysis. The resulting My Hearing PREM was a 16‐item long form or 9‐item short form measure of an individual's experience of their hearing loss, including their experience of clinical support; in the next phase of the HeLP research program these measures were validated and demonstrated to be reliable with good construct validity [14, 15].

We present the final phase and work package of the HeLP research program which aimed to assess the feasibility of implementing the validated My Hearing PREM into routine National Health Service (NHS) audiology service practice. Within the UK NHS there is an increasing requirement for measurement of patient reported experience as part of health service commissioning, for example, in maternity services [16]. This work package was therefore important to address known challenges with implementing both PREMs and PROMS into clinical practice, with common issues including lack of resources, organizational culture, clinicians' beliefs about the value of PREM tools more broadly, and IT challenges [17, 18, 19, 20]. There are also challenges for services as to how to use data to drive quality changes in practice, with common barriers reported to include lack of preparation or consideration as to how data will be used, and lack of time and expertise in data analysis and QI [8, 17].

The specific objectives of each stage of this work package were to:

- Stage 1: Understand the challenges to implementation of the My Hearing PREM into clinical practice from the patient and audiologist perspective.
- Stage 2: Develop implementation resources to support adoption and use of My Hearing PREM in clinical practice.
- Stage 3: Determine the acceptability of the implementation plans and resources from the patient and audiologist perspective.
- Stage 4: Implement My Hearing PREM and evaluate the implementation process in three UK audiology sites.

This is the first investigation of the feasibility of implementing a PREM in audiology, and the insights gained will be relevant for wider implementation of PROMs and PREMs into audiology and other services.

## 2 Methods

### 2.1 Clinical Sites

We worked with three NHS clinical audiology departments, two in England and one in Scotland, to plan, implement and evaluate implementation of the validated PREM into practice. These departments were all publicly funded, and varied in location (urban, sub‐urban and rural), service configuration and population demographics to ensure findings would be applicable across diverse audiology services. They are denoted as sites A, B and C.

### 2.2 Person‐Based Approach

**Figure 1:**
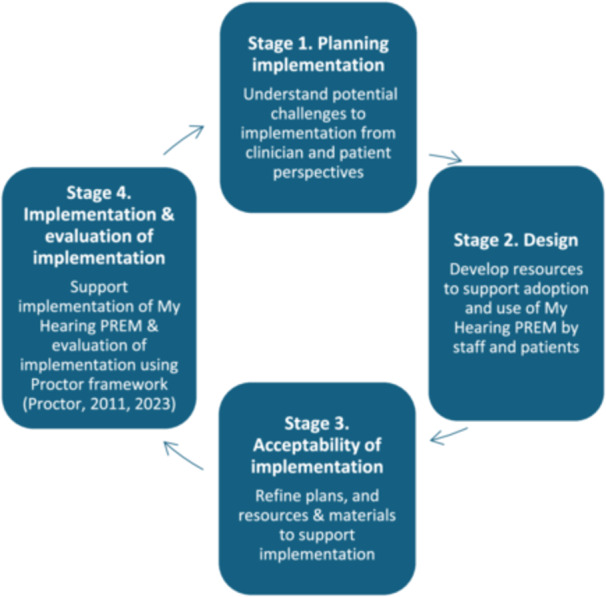
Stages in the Person‐Based approach adapted for my hearing PREM implementation planning and evaluation.

To guide implementation of the My Hearing PREM into practice, we applied the Person‐Based Approach (PBA) [21]. PBA is underpinned by the aim to understand and accommodate the perspective of the people who will use the intervention (where the intervention in this case is the plans to implement My Hearing PREM into practice), and has been evidenced to improve uptake and use of interventions [22]. We applied the PBA framework across the four stages of implementation development: planning, design, acceptability testing, and implementation with evaluation of the implementation process (Figure 1).

PBA draws on qualitative and mixed methods approaches and engages with a wide range of people from the target user group (in this case clinicians and patients). It provides an iterative approach whereby an intervention goes through several development, testing and refinement loops.

### 2.3 Overview of Methods and Data Collection

**Figure 2:**
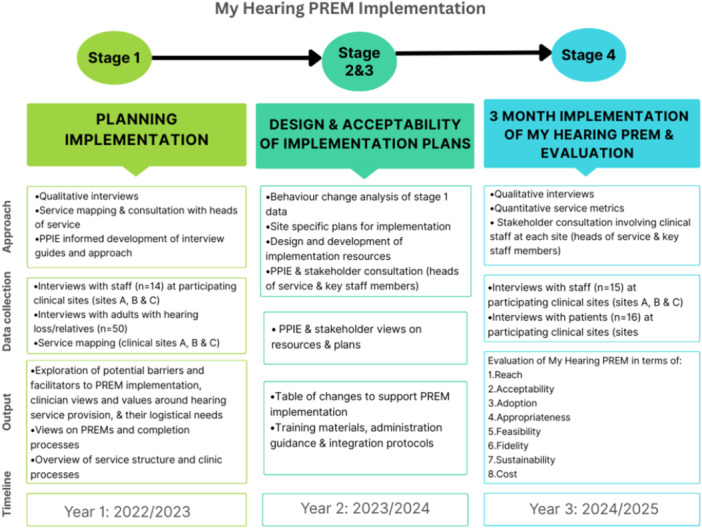
Stages of My Hearing PREM implementation using the person‐based approach.

The work package described in this paper consisted of four sequential stages of work, aligned with the PBA as outlined in Figure 2. The sequence of work relied on methodological pluralism [23] where inductive work used qualitative methods (e.g., to provide granular detail on experience and process of implementation) and quantitative methods were used to describe the 3 month period of clinic activity. This is in line with pragmatist approaches to solving research problems [24]. The four stages took place over a 3 year period (2022–2025), with My Hearing PREM implemented for 3 months of the third year.

For stage 4, the evaluation of implementation was informed by the widely used evaluation framework of Proctor et al [25, 26]. Table 1 summarizes the constructs assessed and the methods of data collection.

**Table 1:** Implementation constructs and specific questions addressed [25].

Implementation construct	Questions addressed	Methods of data collection
Reach	What is the % completed PREM of those that are eligible?	Quantitative: data on use
Acceptability	Do audiologists and patients view the PREM as acceptable, satisfactory, agreeable in practice?	Qualitative: staff and patient interviews
Adoption	Do audiologists use the PREM in their practice?	Qualitative: staff and patient interviews
		Quantitative: data on use
Appropriateness	Do audiologists and heads of service perceive the PREM as relevant and useful to their practice and service management?	Qualitative: staff interviews
Feasibility	Is the PREM suitable for everyday use within the department?	Qualitative: staff interviews
Fidelity	Is the PREM implemented as intended?	Qualitative: staff and patient interviews
Sustainability	Did the use of the PREM change over the 3‐month implementation period? Will the department continue to use the PREM?	Qualitative: staff interviews
		Quantitative: data on use
Cost	How much does it cost to implement the PREM?	Quantitative: data on resource use

The qualitative interviews and PPIE/stakeholder consultation were conducted and analyzed by three female qualitative researchers and one male qualitative researcher. They included one academic‐clinician researcher (a hearing therapist, HP), and three academic researchers with backgrounds in sociology (GBOC), health psychology (SS) and implementation science (JB). The quantitative clinical PREM data were analyzed by two female researchers AH (an audiology clinician researcher) and SN (a health economist). The whole team met regularly through the process to discuss all the analysis, interpretation of findings and to plan how findings at each stage should influence subsequent stages.

Regarding reflexivity, the team brought different perspectives to the project. There were team members with and without hearing loss themselves, and with and without carer responsibility for people with hearing loss. The team included members who worked clinically in audiology as well as those without such experience. This brought different perspectives to implementation planning as some team members were involved in audiology service provision and some were service users. We reflected throughout on these perspectives and were alert and transparent about assumptions we brought based on our personal experiences or roles. We reflected on our impressions of the data and how our perspectives shaped our interpretations of accounts.

### 2.4 Participants and Recruitment

A total of 95 participants contributed data across the four different stages, from planning through to evaluation (further details in Figure 2). Participation was not designed to be longitudinal across stages, and individuals were recruited separately at each stage.

### 2.5 Staff

At stages 1 and 4, staff from the three participating NHS audiology departments were recruited through team meetings and email invitations. All staff were invited to contact the researcher allocated to their site to arrange an interview, with both online and in‐person interviews conducted. Participants represented a mixture of roles including audiologists, hearing aid dispensers, administrative staff, and managers. Number of participants are shown in Figure 2.

### 2.6 Patients, People With Hearing Loss and Caregivers

Patients and people with hearing loss were involved in stages 1 and 4. Eligibility criteria for patient participants included being 16 years or older, living with hearing loss or being a caregiver of someone with hearing loss, having the capacity to provide fully informed consent, and communicating primarily through spoken language. In stage 1, adults with hearing loss who participated in the concept development interviews as part of the HeLP study [13] were asked about their views on PREMs and a hearing‐specific PREM at the end of their interviews about their lived experience of hearing loss.

In stage 4, patients were also recruited via the three clinical sites and included individuals with hearing loss and family members/carers, with some participants having dual roles (being both a person with hearing loss and a carer for someone else with hearing loss). Number of participants are shown in Figure 2.

### 2.7 Qualitative Data Collection and Analysis

#### 2.7.1 Stages 1 and 2: Planning and Design

Semi‐structured interviews were conducted with staff at participating clinical sites focusing on the potential barriers and facilitators to PREM implementation. The interview schedule was informed by PPIE consultation and steering group advice [11, 14] and explored clinician views of a PREM and how it could be incorporated into their workflow, the perceived values of a PREM in hearing service provision, and the logistical needs of introducing the tool (see Table S1 for interview schedule). Patients were also asked about their views on PREMs and the process of completion, providing insight into patient preferences for implementation and their perceived value of the tool and its purpose in a clinical setting.

Interviews lasted up to 1 h and were recorded via MS Teams software with automatic transcription supplemented by manual checking for accuracy. Two researchers (GBOC & SS) conducted the interviews.

Data were thematically analyzed using Framework Analysis [27], a method that allows themes to be explicitly considered a priori in accordance with the research questions and identified through inductive analysis. Four authors (GBOC, JB, HP and SS) read the transcripts to identify themes and develop a coding index. Transcripts were coded by SS and synthesized within thematic matrix charts. Mapping and interpretation involved comparing and contrasting responses across themes to identify explanations for findings.

Particular attention was given to how the PREM could be incorporated into clinical workflow and how barriers to implementation could be overcome. Drawing on theoretical behavior change theory such as the behavior change wheel [28] and the taxonomy of behavior change strategies [29], we created a table of changes needed to support the implementation of the PREM (see Table 2).

**Table 2:** Key concerns and views on benefits of using a PREM from both staff and patient perspectives.

Views on concerns and benefits	Illustrative quotations	Task for design of implementation materials
Concern: Existing clinical practices may duplicate the information gained from the PREM	*“And so I think we're quite good for the most part, getting that information from patients but still think we could probably do better to get those sorts of qualitative values and gap in the more sort of quantitative view” (Audiology clinician)*	For audiology teams: explain the unique purpose of the PREM in supporting the case history process
Concern: Questioning the value of questionnaires as a clinical tool	*“Questionnaires can sometimes be quite quantitative. You know, is your experience a 2 or a 3 out of 10 and it's like, well, I don't know, but that's not the point really, and I think that I like qualitative and openness would probably be more valuable than like. What feels like an exam or a metric based kind of questionnaire?” (Audiology clinician)*	For audiology teams: explain the purpose of a validated PREM as a way of measuring experience both within and between patients
Concern: Staff and patients unfamiliar with the notion of patient reported experience measures	*“Well, I've only heard that term yesterday, but obviously I'm very familiar with PROMs which is the outcome measure bit.…there's not a lot of them around, is it?” (Audiology clinician)*	For audiology teams: explain purpose of PREMs For patients: explain the purpose of the PREM
Concern: Additional tasks required in completing a PREM	*“So within the audiology appointments, they're all quite formulaic, as in there are certain things that they have to do. So you know, in a hearing aid assessment, they've got their own questionnaires. They've got the hearing test to do. They've got the talk through of the test results and then decision as to whether or not you have a hearing aid… I guess for them, adding something else in might feel a bit difficult for them.” (Audiology clinician)*	For audiology teams: consider and discuss best ways of administrating the PREM locally
Concern: Questioning whether the PREM was suitable for certain groups of people, e.g., those cognitive or learning difficulties; teenagers	*“Obviously, a lot of our patients are quite elderly, so if that maybe they're having memory problems or maybe they're quite tired and clinic and you're maybe struggling to get accurate hearing test results, never mind anything else, I think you would probably struggle or struggled to want to do it in that sort of situation” (Audiology clinician)*	For audiology teams: explain that the development process of the PREM involved and was tested with a wide range of people included those groups mentioned
Concern: Not having knowledge of, or access to additional referral pathways	*“I know there are like lip reading classes and but I don't know if there's really much out there in the way of I just don't know about it, but support groups” (Audiology clinician)*	For audiology teams: ensure staff are aware of wider support and referral pathways
Concern: Patients querying whether audiologists have the time, skills or willingness to engage with their responses	*“they seem to have been trained very much to do with the technology and not to do with the social aspects of hearing loss so, and we seem to have very little awareness of how it impacts people socially, which is a massive issue” (Patient)*	For audiology teams: explain the need to engage with the patients’ PREM responses
Concern: Questions about when in the patient pathway to administer	*“I think it's important that if that questionnaire was sent with appointments so people with hearing loss or issues, experiencing hearing issues of any kind, that they got to write their answers to it because it's not until you sit back and think, like I said you take for granted what you have, so it's not until you sit back and think “well hang on a minute, yes it does impact me”. (Patient)*	For audiology teams: advise that the PREM is best sent to patients in advance of their appointment For patients: explain the procedure for filling in the PREM and discussing with their audiologist
Benefits: Measuring what matters	*“So I think if we're sort of considering the experiences of a patient's hearing loss and we want to know more about that, I definitely want to know about what they think about our service and the way that it's running, if there are any improvements that we can do” (Audiology clinician)*	For audiology teams: Emphasize the value of capturing significant content to the clinical encounter
Benefits: Create opportunities to target care	*“I think it it's a good opportunity to kind of open up conversation with the audiologist. Other than the generic chit chat. Umm and yeah, just could get the person to maybe elaborate on what they've said” (Patient)*	For audiology teams: Emphasize the opportunity of the PREM to tailor care
Benefits: A tool for reflection	*“I think it opens up the opportunity for the patient to then and maybe explain why they're feeling. Uh, so why or why they've had certain experiences and it kind of I think will help to open up that opportunity for the conversation to take place” (Audiology clinician)*	For audiology teams: Emphasize the opportunity of the PREM to explore wider issues and consider all aspects of the impact of hearing loss

#### 2.7.2 Stage 3 and 4: Acceptability and Evaluation of Implementation

Rapid qualitative research methods were used involving a series of focused questions to interview participants [30]. This approach was chosen at these later stages to enable timely data collection within the short timeframe available for implementation of My Hearing PREM (i.e., three months). As the aim of these interviews was to examine the feasibility of using My Hearing PREM in practice, the questions were specifically focused on the experience of implementation. In line with other evaluation studies, rapid qualitative work allows for targeted examination of topics, combining inductive views with focused questions and collation of responses into rapid assessment sheets [31].

Interviews lasted under 30 min (see Table S1 for interview schedule). With participants' consent, the interviews were audio‐recorded using a digital voice recorder or MS Teams. Field notes were taken to note information not captured by the recordings.

We drew on a rapid qualitative analysis approach informed by Framework analysis [32]. This analysis differed to Stage 1 as it used a template based on the interview questions for rapid analysis (whereas Stage 1 was more in‐depth and used a framework developed from the inductive analysis). Following each interview, the researchers (GBOC and SS) rapidly summarized data within 24 h using transcripts generated by voice recognition software and field notes, allowing immediate analysis. This rapid summary was completed by the researcher based on their immediate impressions, whereby instead of generating standalone summaries first, the team directly populated matrices structured by pre‐defined categories guided by the interview topic guide in MS Excel. These matrices, organized by participant rows and thematic columns, enabled quick comparison across cases. For each participant, data were summarized across each category identified in the matrix. Illustrative or insightful quotes were selected to support and demonstrate the interpretation for each theme. Authors (G.B.O.C., H.P., S.S.) met regularly to facilitate iterative analysis and consistency between researchers. A PPIE representative was consulted throughout to ensure patient perspectives were accurately reflected in our interpretations.

### 2.8 Quantitative Data Collection and Analysis

#### 2.8.1 Stage 4: Evaluation of Implementation

Clinical data stored on the electronic management system of each site were extracted by the individual departments to answer the questions relating to adoption, reach and sustainability of the My Hearing PREM (Table 1).

The costs of implementing the My Hearing PREM were based on estimates of staff time and resources that would be required to implement the PREM in practice, the values of which were obtained from the three audiology services and the research team. They included staff time in planning of the PREM implementation; time to set up the PREM into the electronic patient record; staff training including the time of the facilitators. Travel time and costs of the facilitators were based on those of one local center, as this was deemed to be closer to how the PREM training would be implemented in practice. No time was allocated for the use of the PREM within the audiology clinics as the PREM was integrated into current appointment slots. The costs of printing the PREM were also included. The time of each different clinical staff grade was valued using the latest version of Unit Costs of Health and Social Care [33]. Research team time allocated during the current study was valued using University of Aston salary scales and included overheads.

### 2.9 Patient and Public Involvement and Engagement

Patient and public involvement and engagement (PPIE) were integral to all stages of the implementation planning work. A PPIE representative with lived experience of hearing loss was recruited to the research team and contributed throughout the HeLP study (see [11]). Their involvement included contributing to the development of interview schedules, reviewing data analysis interpretations to ensure findings reflected participants' experiences, and providing guidance on the development of implementation resources. The PPIE representative participated in data analysis sessions with the research team, helping to check interpretations and ensure patient perspectives were represented in our analysis of the data. This collaborative approach ensured that the implementation planning remained grounded in the real‐world experiences and need of people with hearing loss.

## 3 Ethics Approval

The study was approved by the West of Scotland Research Ethics Service (approval date: 6 May 2022; ref22/WS/0057) and the Health Research Authority and Health and Care Research Wales (HCRW) (approval date: 14 June 2022; IRAS project ID: 308816).

## 4 Results

### 4.1 Stage 1: Planning Implementation

Staff and patient views on PREMs are reported according to the key findings that informed the design of implementation materials in stage 2. The key concerns and views on the benefits of using a PREM are summarized in Table 2. The main perceived benefits were focused on the potential to expand the focus of the clinical appointment to address what mattered to the patient.

### 4.2 Stage 2 and 3: Design and Acceptability of Implementation

The findings from stage 1 (summarized in Table 2) were used to inform the design of the implementation materials and the implementation plans for each clinical site.

One of the key findings from stage 1 was the need to differentiate My Hearing PREM so it was not seen as ‘just another questionnaire’ by clinicians. We focused on the potential to use My Hearing PREM as a tool for starting a conversation with patients. This approach was possible because it wasn't a service orientated questionnaire, it was there to expand the remit of the consultation and to identify issues of most importance to the patient. We also recognized that there were concerns about time. We focused on the potential for My Hearing PREM to save time for clinicians because it could identify the key patient issues more rapidly.

These findings were incorporated into a set of resources, developed alongside strategies for implementation (Table S2). These were tailored to each site in collaboration with heads of service and key staff members, and were made available to staff across all sites in advance of implementation of My Hearing PREM (both versions accessible in Smith et al [14]). PPIE and stakeholders gave feedback on the acceptability of these resources, and they were reviewed and adapted as required.

The implementation plan for My Hearing PREM was discussed and agreed with each site individually. A summary of the key elements of each site's implementation plan is shown in Table 3.

**Table 3:** Site specific plans for implementation of My Hearing PREM.

	Site A	Site B	Site C
PREM version used	9‐item	9‐item (audiology appointments) 16‐item (hearing therapy appointments)	16‐item
Eligible appointment types	Adult hearing assessment appointments	Adult hearing assessment and reassessment appointments Hearing therapy appointments (not including appointments for tinnitus)	All hearing related assessment appointments Follow up appointments if appropriate
Method of delivery	Patients asked to complete the PREM in the waiting room before their appointment If not completed in advance, the audiologist to complete during the appointment	PREM sent on the back of appointment letter for patients to complete in advance If not completed in advance, the audiologist to complete during the appointment	PREM sent on the back of appointment letter for patients to complete in advance If not completed in advance, the audiologist to complete during the appointment
Mandated or optional	PREM was optional for staff to use	PREM was mandated for all staff to use if they had time	PREM was mandated for all staff to use if they had time
Additional time or resources provided to staff	None	None	None
Data storage	PREM integrated into the clinical history form on the EPMS	PREM set up as a new custom questionnaire on the EPMS	PREM set up as a new custom questionnaire on the EPMS
Information technology support	Modifications to the EPMS and data reporting carried out in‐house	Modifications to the EPMS and data reporting outsourced	Modifications to the EPMS completed in‐house Data reporting outsourced
Implementation period	3 months	3 months	3 months

Abbreviation: EPMS, electronic patient management system.

### 4.3 Stage 4: Evaluation of Implementation

The implementation process in each site was evaluated according to the set of implementation constructs using both quantitative and qualitative methods of data collection (summarized in Table 1). Quantitative data are reported as ranges or trends to protect the anonymity of the sites in Table 4.

**Table 4:** Summary of quantitative evaluation data.

Construct	Finding
Reach: PREM completed as a percentage of eligible patient appointments	Audiology appointments: 17%‐27% across sites A, B and C Hearing therapy appointments: 48% at site B
Adoption: Do audiologists use the PREM in practice?	There was variation in use across all audiologists. In each site, there were audiologists who used the PREM in over 50% of their eligible patients, and others who used it in less than 10% of patients or not at all
Sustainability: Did the use of the PREM change over the implementation period?	In two sites, there was a reduction in use over the 3‐month implementation period. In the other site, usage was more stable.
Cost: How much does it cost to implement the PREM?	Costs varied by site, between £4365 and £6239, and between £1.57 and £3.18, on a per patient basis Factors that influenced the variation in cost included the number of planning meetings required, the number of staff involved in planning, and amount of time required for each site to set up and integrate the PREM into their electronic patient management system (varied from 4–15 h)

### 4.4 Acceptability and Appropriateness

Acceptability refers to whether the PREM is agreeable or satisfactory; appropriateness, a related construct, is the perceived fit for a particular problem or setting. These are related constructs and are discussed in combination.

From the clinician perspective, My Hearing PREM was seen as a tool that could access the lived experience of patients, their challenges and social frustrations. Clinicians felt the PREM encouraged patients to reflect on their situation and experiences and had the potential to empower a proactive approach to their condition.

It's got patients thinking. About it in a more maybe I don't know if realistic is the right word, but maybe in a more idea of how it's actually affecting them, maybe more from a personal emotional side rather than just a functional side.

(Audiology clinician)

It was agreed that posting the PREM in advance to patients to complete was better than filling out in clinic, as this gave the patient time to reflect on areas of their life that they might not consider are related to hearing issues and gave the clinician information which could inform the direction of the consultation.

There were concerns about some sections of the PREM. Clinicians felt more comfortable talking with patients about the communication questions than the section on support. Clinicians were concerned about questions that were focused on health professionals, as they felt this could be used for monitoring their performance, or were difficult for patients to answer when they hadn't yet had the experience to draw on. There was uncertainty about the scoring system, how it should be used and how the data generated were used.

I think couple of questions may not be appropriate for them to complete before their appointment like “I'm aware of what hearing test involved”… All the new patients, they sort of they never had hearing tests, they don't really know what it involves until they have come to the department.

(Audiology clinician)

Clinicians raised concerns about the appropriateness of the PREM for some patients, such as those who need more time. Clinicians saw aspects of the PREM as duplicating their practice, such as the history taking, whereas others highlighted it could be useful to take a more detailed history.

Useful for struggling to get a good history from the patient, this can encourage them to open up more.

(Audiology clinician)

Patient participants were asked to reflect on their experiences of using the PREM following its introduction into routine appointments. Patients generally reported that they had a clear sense of purpose when attending their appointments and felt that the PREM aligned well with this. For many, completing the PREM helped them become more aware of what they were experiencing, particularly in terms of how hearing loss was affecting different areas of their life. Although not the case for all participants, several participants described the PREM as a useful prompt for thinking through what they wanted to discuss with their audiologist.

So it's upsetting writing that down because it's visually pointing out the things I go through each day. But it is making me more confident saying, no, you know, I can't hear you. You need to say it again and repeat. And I wear a badge at work as well now, which is what I done.

(Patient)

Most patient participants found the PREM easy to complete, including those with additional needs such as early‐stage dementia, language barriers, or learning difficulties. The questions were generally seen as appropriate and relevant, with many noting that the wording reflected their own experiences and concerns. That said, not all participants felt equally confident when completing the questionnaire. A few expressed uncertainties about how to choose the “right” response, especially when they felt their experiences did not fit neatly into the response options. This hesitancy did not appear to affect overall engagement with the PREM but does point to the importance of providing clear instructions and reassurance that there are no right or wrong answers.

So that was correct. Doing that. Yes, right, that, that's what was confusing out, and didn't know whether I was putting the right thing down. We didn't, right. Yes. OK all right.

(In the interview, the patient went through some questions to check his choice of response matches what he thinks and feels)

### 4.5 Adoption

The quantitative data showed a relatively low uptake of the PREM within each site, and variation in use in each site across audiologists. Within each site there were clinicians who used the PREM very little, to those who used it with over half to most of their patients. For some the PREM was optional and when consultation time was seen as limited, clinicians made the decision not to complete the questions with patients.

Did it at the end because it is not the priority of the appointment and so can only fit it in if enough time left at end of the appointment.

(Audiology clinician)

### 4.6 Feasibility

Feasibility refers to how successfully the PREM can be used in the setting. In terms of efficiency, all services found it beneficial if patients completed the PREM in advance, either at home or in the waiting room. However, not all patients brought a completed PREM into the consultation which limited its chance of being used in the appointment.

Sometimes if the patient doesn't give it [PREM] to me and we just launch into the appointment as sometimes happens, I sometimes forget to say, oh, did you, did you bring that?

(Audiology clinician)

Issues relating to IT and electronic storage of the PREM had a significant impact on feasibility across all three sites. These problems were generated from the addition of the PREM as a new questionnaire to the audiology EPMS. They included a reduction in the speed of the database impacting on workflow, lack of automated scoring of the PREM, and poor user interface and functionality.

### 4.7 Fidelity

This construct examined whether the My Hearing PREM was implemented as intended. There were some indicators that the discussions generated by the PREM impacted on clinical decision making, including signposting to other services, but clinicians were not always able to identify specific examples. Several patient participants noted that the questions resonated with their lived experience and encouraged them to think more critically about their needs, which in turn helped them feel more prepared for their appointment.

I found several of them a little bit difficult, partly because I'm in some ways new to this because my and I think not really a negative thing, but it's helped me realize that I do have a hearing problem and to acknowledge that to myself. As I do acknowledge that sometimes with friends or with other people you know, saying I'm sorry, I don't hear very well

(Patient)

At the same time, the process of completing the PREM offered insight into how well the tool worked in real‐world settings. While most patients were positive about the experience, some noted that their clinician didn't respond to their answers within the session.

She entered the answer in the relevant bits on the screen and OK, so it's no was there any sort of discussion or not really no. I mean the questions were clear and I gave the best answer I could and she put it in there. Yeah. There wasn't really much discussion, I don't think

(Patient)

### 4.8 Sustainability

All sites reported that they are going to keep My Hearing PREM as an optional tool for individual clinicians to support clinical practice, but no sites plan to use it at as standard at a service level to gather and report on patient experience. One reason for this was the lack of a mandate for audiology services to report on meaningful patient experience data to commissioners, and a lack of national audits, such as those conducted in other speciality areas like Parkinson's disease.

Alarming lack of reporting other than diagnostics

(Audiology clinician)

## 5 Discussion

This study explored the process of implementing My Hearing PREM into routine clinical practice across three NHS audiology departments. There is increasing recognition that patient experience is a crucial component in healthcare quality, linked to safety and clinical outcomes [4, 34]. Our findings support the idea that tools like the My Hearing PREM can help embed this ethos into audiology services whilst highlighting the complexity of translating the use of a PREM into everyday practice.

There was clear evidence that My Hearing PREM enabled some clinicians to develop a more holistic understanding of their patients' experiences and acknowledge the social and emotional dimensions of hearing loss. This reflects the original purpose of My Hearing PREM which was to center patients' lived experiences, rather than focus solely on their clinical presentation [13]. The tool prompted more reflective conversations and appeared to promote patient‐centered interactions in some instances. This is encouraging and suggests that My Hearing PREM could have a role in supporting audiologists to move their communication style to become more patient‐centered [35]. Completing the My Hearing PREM often functioned as a reflective exercise for patients, allowing them to consider what they most wanted to communicate during their appointments. This may be particularly useful for individuals who had not considered the personal impact of hearing loss before or found it difficult to articulate this during (time‐limited) clinical encounters. All three sites have opted to retain the My Hearing PREM as an optional resource, suggesting that it was perceived as useful. There was a low cost to implementing the PREM, and the cost per patient would decrease over time if use became embedded in a service.

Despite these promising signs, implementation was uneven. Uptake varied both across and within sites, with some clinicians engaging regularly with the measure, while others rarely used it. This variation aligns with wider implementation literature, which emphasizes that engagement is shaped by perceptions of a tool's relevance, ease of use, and compatibility with existing workflows [17, 18, 19, 20]. Some clinicians questioned whether the PREM added value, particularly where its content overlapped with questions they already asked. Others were uneasy about the evaluative nature of some questionnaire items, especially those relating directly to their own practice. Clinicians' varied engagement and concerns about PREMs as evaluative tools [18, 36] suggest that implementation approach and organizational messaging may be critical factors in determining whether these measures are perceived as collaborative improvement tools or audit mechanisms. Without clear system‐level support and communication about the purpose of the PREM, clinicians may view it through a more skeptical lens, limiting its potential to support quality improvement and patient‐centered care.

There was a recurring feeling of uncertainty about how the My Hearing PREM data would be used. Patient participants were unsure where the data went, who reviewed their responses if they were reviewed, or whether completing the My Hearing PREM made any difference to the care received. For clinicians, this uncertainty made it harder to prioritize the tool in time‐pressured settings. These findings align with concerns that PREMs are sometimes implemented without adequate planning for how to translate data into action [8, 37]. Clinicians may perceive such tools as burdensome rather than beneficial to their practice when feedback loops are unclear or incomplete. This can be addressed through regular reporting of scores back to clinicians in ways that are meaningful to them [34].

Clinicians generally favored sending the PREM to patients before appointments, which allowed patients to complete it in their own time without influencing their clinical time too much. However, pre‐appointment completion by patients was inconsistent, and this influenced whether the clinicians used it within the appointment. Some clinicians felt unprepared to respond to unexpected responses raised by patients or issues requiring more time.

Technical barriers also limited implementation. Across sites there were challenges in meaningfully integrating the My Hearing PREM into the electronic patient management system used in NHS Audiology departments, which posed a significant barrier to adoption. There were technical difficulties such as challenges with data handling, data storage and uncertainty around scoring. Some staff had hoped for practical features such as real‐time scoring or decision‐support prompts, but these were not possible within current IT infrastructure. The inability to (easily) extract and report on the data meant that clinicians and service leads lacked access to feedback loops, making it difficult to demonstrate the tool's value and removing the potential to use the information for service improvement. This disconnect between the PREM and existing digital infrastructure limited its visibility within clinical pathways and perhaps undermined its potential as a routine part of care within the sites. This is similar to other studies citing technical infrastructure as a barrier (see [38]).

Both patients and staff raised concerns about whether there was scope to act on some of the issues identified. This raises important questions about the remit of audiology services and whether tools like My Hearing PREM may reveal patient needs that extend beyond what current provision is designed to address. Differences were observed in engagement with the PREM between audiology and hearing therapy teams. Hearing therapists appeared more open to using the PREM, perhaps because their roles are more closely aligned with the psychosocial aspects of care. These findings highlight the importance of context in implementation, suggesting that measures have the potential to be more easily embedded within a service when they align with the service's ethos and aims. They also encourage consideration about the scope of audiology practice and whether tools like the My Hearing PREM could reveal patient concerns or experiences which current services are not organized or structured to manage, for example, social isolation as a result of hearing loss.

While the My Hearing PREM was generally viewed as valuable, it was not possible to measure whether or how its use directly influenced care. Next stage research is needed to evaluate how PREMs could actively shape care delivery and outcomes, beyond serving as a measure of experience which prompts reflection.

### 5.1 Strengths and Limitations

A key strength of this study was the staged approach to the planning and implementation of the My Hearing PREM into the three clinical sites. The tool itself was co‐produced with ongoing input from public and patient involvement and engagement (PPIE) contributors, ensuring it was informed by lived experience from the outset [13]. This included input from a diverse range of individuals, such as those with dementia, learning difficulties, and those who speak English as an additional language. Clinicians were also involved as stakeholders throughout the development and implementation phases, helping to ensure the tool was grounded in what happens in everyday routine audiology practice.

Trialing My Hearing PREM in real‐world settings across three NHS audiology departments added to the ecological validity of the findings. The reflexive and iterative approach to implementation where early feedback was used to refine later strategies and resources also contributed to the relevance and sensitivity of the work.

An intentional feature of the implementation strategy was its non‐prescriptive approach to implementation, allowing flexibility in how and when the PREM was used across the different sites. This approach enabled services to tailor implementation to their local context. However, it also resulted in variability and inconsistency, both between and within sites. This raises questions about the balance between flexibility and fidelity, an issue discussed within implementation science literature [39, 40]. While adaptability can support uptake in diverse settings, it can also influence standardization and hinder our ability to compare outcomes across services.

Much of the qualitative data from the planning stage of implementation reflected clinicians' anticipated views, as the PREM had not yet been finalized or fully introduced. In some cases, uncertainty may have stemmed from a general unfamiliarity with PREMs and their role in audiology care.

Whilst the specific findings reported here are unique to this evaluation, broader points about using established implementation models and pluralist approaches to evaluation may be transferable to other contexts.

## 6 Conclusions

This study indicates that My Hearing PREM can support more personalized, reflective care in audiology by bringing patient experience to the fore. The person based approach was a useful model to plan and support implementation. The PREM was introduced across three NHS audiology sites and clinicians were generally open to using it. However, use varied and integration into routine practice was inconsistent. Its long‐term use and impact will depend on tackling both practical barriers such as time constraints, IT limitations, and lack of clarity around what PREMs are, and how to use and act on the data. More broadly, it will be important to address clinician concerns about the purpose of PREMs and whether they align with services' existing ways of working.

## Author Contributions

**Amanda Hall:** conceptualization, data curation, formal analysis, investigation, methodology, writing – original draft presentation, writing – review and editing. **Helen Pryce:** conceptualization, formal analysis, funding acquisition, investigation, methodology, project administration, writing – original draft presentation, writing – review and editing. **Georgina Burns‐O'Connell:** data curation, formal analysis, investigation, methodology, project administration, writing – original draft presentation, writing – review and editing. **Sian Smith:** data curation; formal analysis, investigation; methodology, project administration, writing – review and editing. **Sian Noble:** conceptualization, formal analysis, methodology, writing – review and editing. **Jon Banks:** conceptualization, formal analysis, methodology.

## Ethics Statement

The study was approved by the West of Scotland Research Ethics Service (approval date: 6 May 2022; ref22/WS/0057) and the Health Research Authority and Health and Care Research Wales (HCRW) (approval date: 14 June 2022; IRAS project ID: 308816).

## Conflicts of Interest

The authors declare no conflicts of interest.

## Supporting information


Supporting File 1

Supporting File 2

## Data Availability

The authors have nothing to report.
