# Supplementary material for: Implementation of My Hearing PREM Into Three UK Audiology Services: A Pluralist Approach to Planning, Design and Evaluation
Source: Health Expect. 2026 Apr 11;29(2):e70659. doi: 10.1111/hex.70659 (PMC13069358; doi:10.1111/hex.70659)
Supplement: Supplementary file 1 — Supporting File 1 [file HEX-29-e70659-s001.docx]

*Supplementary table 2: Interview schedule*

| **Stage 1** | |
| --- | --- |
| Clinicians | - How do patients share their experiences of hearing loss and hearing aid use with you? - How familiar are you with PREMs? (Gauge understanding, have they used something similar before, familiar with the term/ concept of measuring patient experience) - In your current role, how useful would the PREM be for you? Why/ why not? - How useful would the PREM be for patients? (prompt- any particular groups who would/ would not complete it, extent which it would overburden)? - What would you hope for from a measure of patient experience? (prompt- what would you want it to achieve? What would you want it to look like – maximum number of items, sections) - How feasible would it be to routinely administer a PREM tool in your service? - How would you talk and explain about this tool to patients? (prompt- how would you describe and introduce it to patients?) - What do you see as the best way to deliver/ implement the PREM? (prompt- which staff might be involved to deliver tool? When could it be administered – at what stage? Before, during or after consultations?) - How would you prefer the tool to be administered –paper-based or electronic based (online)? (prompt- clinician’s view and also perceptions of what they think patients would prefer)? - What do you see as the main barriers to using the PREM if it was introduced into practice? - What do you see as the main facilitators to using the PREM it was introduced into practice? - what kind of resources would help clinicians work with the PREM and how they might introduce the PREM to patients e.g. videos, leaflets, weblinks to online guides |
| Patients | - PREM- aim to develop questionnaire to capture patient experience that patients would fill in. Audiologist would know how to aim to improve knowledge of patient experience of hearing loss. People with hearing loss adapt communication and navigate hearing services (audiology). We have designed a way to show whether the services are working well for their patients. - What are your thoughts on filling this in? - How helpful would this type of questionnaire be/ - When do you think the questionnaire should be filled in (consultation with clinician, at home)? - Would you prefer to complete it online, paper based? - Can you tell me a bit more about..? |
| **Stage 4** | |
| Clinicians | - Perception of training and implementation resources - How does PREM fit within normal work patterns - Do the appointments take the same time, less time or more time. Quantifying by 5 minute increments - How easy/long/time taken to input into electronic records (any questions left blank?) - Perception and use of the PREM (acceptability, adoption, appropriateness) - For patients - For the service - Barrier or facilitator to clinical conversations - Changes made to incorporate PREM into clinics - Impact and changes to consultation and referral outcomes - For heads of department: Views on the service improvement plans informed by the PREM and the proposed service reconfiguration (sustainability) |
| Patients | - Did you complete the questionnaire at home, in the waiting room or with the audiologist? - If posted, and did not complete at home, ask why? - Did you have any help filling it in/ answering the questions? (clinician, family member) - How did you find completing the questionnaire? - How helpful was it in thinking about your hearing? - Did it help you think about things you hadn’t considered before regarding your hearing? - Did your audiologist discuss your answers? - Do you think it helped the audiologist understand your hearing needs/experience better? - Did completing the questionnaire help talking about your hearing? - Were you given any advice / information based on your answers? - Did your audiologist recommend anything specific after seeing your answers? - Is there anything else you’d like to share about your experience? |
