# Supplementary material for: Implementation of My Hearing PREM Into Three UK Audiology Services: A Pluralist Approach to Planning, Design and Evaluation
Source: Health Expect. 2026 Apr 11;29(2):e70659. doi: 10.1111/hex.70659 (PMC13069358; doi:10.1111/hex.70659)
Supplement: Supplementary file 2 — Supporting File 2 [file HEX-29-e70659-s002.docx]

*Supplementary table 2: Implementation strategies*

| **Strategy** | **Target audience** | **Developed & delivered by** | **Content** | **Adaptive** |
| --- | --- | --- | --- | --- |
| Introductory meeting | Clinicians & managers | Research team | Introduction to the learning materials & implementation strategies, infographics & film | Yes |
| Film | Clinicians | Research team & PPIE | What is a PREM?  How different to a PROM? | No |
| Process infographic | Managers  Clinicians | Research team & PPIE | Overview of the implementation process | Yes |
| Process infographic | Clinicians | Developed by research team, clinical team & PPIE; delivered jointly by HoD/research team | 1 page “how to” guide tailored to each site | Yes |
| Process video | Clinicians | Developed jointly by research team & HoD | “How to” video guide tailored to each site | Yes |
| Film | Clinicians | Research team & PPIE | “How will the PREM help me?” | No |
| Launch | Clinicians | Delivered jointly by HoD/research team | Team session | Yes |
| Information sheet | Patients | Research team & PPIE | “How will the PREM help me?”  Link to HeLP website | No |
| Refresher meeting | Clinicians | Delivered jointly by HoD/research team | Any specific issues to address | Yes |
| Team meeting | Clinicians  Manager | Delivered jointly by HoD/research team | Reflection | Yes |

*HoD: head of department*
